# Nomograms incorporating genetic variants in BMP/Smad4/Hamp pathway to predict disease outcomes after definitive radiotherapy for non‐small cell lung cancer

**DOI:** 10.1002/cam4.1349

**Published:** 2018-05-09

**Authors:** Ju Yang, Ting Xu, Daniel R. Gomez, Xianglin Yuan, Quynh‐Nhu Nguyen, Melenda Jeter, Yipeng Song, Ritsuko Komaki, Ye Hu, Stephen M. Hahn, Zhongxing Liao

**Affiliations:** ^1^ Department of Radiation Oncology The University of Texas MD Anderson Cancer Center Houston Texas 77030; ^2^ The Comprehensive Cancer Centre of Drum Tower Hospital Medical School of Nanjing University & Clinical Cancer Institute of Nanjing University Nanjing 210008 China; ^3^ Department of Oncology Tongji Hospital Huazhong University of Science and Technology Wuhan Hubei 430030 China; ^4^ Department of Radiation Oncology Yuhuangding Hospital Zhifu, Yantai Shandong 264000 China; ^5^ Arizona State University Mesa Arizona 85212

**Keywords:** Hepcidin, nomogram, NSCLC, outcome, polymorphism, radiotherapy

## Abstract

Hepcidin is crucial in regulating iron metabolism, and increased serum levels were strongly linked with poor outcomes in various malignancies. Thus, we investigated if genetic variants in the BMP/Smad4/Hamp hepcidin‐regulating pathway were associated with outcomes in patients receiving definitive radiotherapy for NSCLC. Subjects were 664 NSCLC patients who received ≥60 Gy radiotherapy for NSCLC retrospectively identified from a single‐institution database. Potentially, functional and tagging single nucleotide polymorphisms (SNPs) of *BMP2* (rs170986, rs1979855, rs1980499, rs235768, and rs3178250), *BMP4* (rs17563, rs4898820, and rs762642), *Smad4* (rs12456284), and *Hamp* (rs1882694, rs10402233, rs10421768, and rs12971321) were genotyped by TaqMan real‐time polymerase chain reaction. Cox proportional hazard's analyses were used to assess potential influences of SNPs on overall survival (OS), local‐regional progression‐free survival (LRPFS), progression‐free survival (PFS), and distant metastasis‐free survival (DMFS). Nomogram of each endpoint model was developed using R project. The median patient age was 66 years. Most (488 [73.2%]) had stage III NSCLC. Age, disease stage, receipt of concurrent chemotherapy, and gross tumor volume were independent factors of OS. *Hamp* rs1882694 AC/CC genotypes were associated with poor OS, LRPFS, PFS, and DMFS in multivariate analyses. Besides, *BMP2* rs1979855, rs3178250, and rs1980499 associated with PFS; *Hamp* rs10402233 and *BMP2* rs1979855 associated with LRPFS; *BMP2* rs3178250 associated with DMFS after adjustment for clinical factors. After adding SNPs to each model, all the likelihood ratios were increased; the nomograms were improved significantly to predict LRPFS (*P *<* *0.001) and PFS (*P *<* *0.001), and marginally to predict OS (*P *=* *0.056) and DM (*P *=* *0.057). Our nomograms incorporating significant SNPs in the BMP/Smad4/Hamp hepcidin‐regulating pathway could improve the prediction of outcomes in patients given definitive radiotherapy for NSCLC. Intensified follow‐ups would be recommended for patients with unfavorable outcomes identified in nomograms. Due to the rapid developments of targeted therapies and immunotherapies for NSCLC, it is necessary to further validate our findings in patients receiving such treatments.

## Introduction

About 80% of patients with nonsmall cell lung cancer (NSCLC) are with disease that is inoperable owing to local advancement or distant metastases at diagnosis 1. Definitive radiotherapy is routinely used for unresectable locally advanced NSCLC either as therapy given concurrently or sequentially with systemic therapy or as primary curative therapy without any other surgical or drug therapy. However, treatment outcomes are still unsatisfactory and heterogeneous due to poor response rates, high rates of distant metastasis, and recurrence 2. The heterogeneous outcomes are also complicated by the involved genetic backgrounds. A “one‐size‐fits‐all” approach to treatment is no longer optimal, and easily accessed markers are needed for tailoring treatments to individuals.

The hormone hepcidin was initially identified through a search for novel antimicrobial peptides but was found in 2001 to participate in regulating iron metabolism. Induction of hepcidin synthesis by the liver reduces iron export from macrophages, enterocytes, and hepatocytes 3. Iron metabolism is known to be dysregulated in several types of cancer, and iron overload has been linked to the tumorigenesis in lung cancer. Targeting metabolic pathways of iron may provide new tools for lung cancer prognosis and therapy 4, 5. Hepcidin was mainly synthesized in the liver, and the expression of hepcidin can be also found in tumor cells including breast cancer, lung cancer, and prostate cancer 6, 7, 8. The increased expression of hepcidin‐induced iron retention can promote the proliferation of tumor cells 8. Increased expression of hepcidin was found to correlate with poor outcomes of patients with breast cancer or renal cell carcinoma 9, 10. High serum hepcidin levels have also been linked with lymph node metastasis and clinical stage in NSCLC 7. Our preliminary work has suggested that expression of hepcidin correlates with and is predictive of overall survival (OS) in patients with NSCLC (in review).

Hepcidin expression is regulated in part by bone morphogenetic protein (BMP) signaling 11. Signals from BMPs act through type II and type I serine–threonine kinase receptors to phosphorylate downstream Smad1/5/8, which then bind to the common mediator Smad4 to regulate the transcription of *Hamp* 12. Mutations in genes regulating hepcidin could induce iron‐deficiency anemia 13, which is thought to cause hypoxia‐induced resistance to radiation in tumor cells. To the best of our knowledge, no studies have investigated potential associations between single nucleotide polymorphisms (SNPs) in hepcidin regulatory BMP/Smad4/Hamp signaling pathways and outcomes (disease control and survival) among patients with NSCLC receiving definitive radiotherapy. To address this gap, we selected 13 potentially functional and tagging SNPs in *BMP2* (rs1979855, rs170986, rs1980499, rs235768, rs3178250), *BMP4* (rs4898820, rs762642, rs17563), *Smad4* (rs12456284), and *Hamp* (rs1882694, rs10402233, rs10421768, rs12971321), hypothesizing that these SNPs in hepcidin regulatory BMP/Smad4/Hamp would be correlated with outcomes among patients with NSCLC after definitive radiotherapy. We defined outcomes in terms of overall survival (OS), local‐regional progression‐free survival (LRPFS), progression‐free survival (PFS), and distant metastasis‐free survival (DMFS). Moreover, our study aimed to develop predictive nomograms incorporating significant clinical characteristics and genetic variants for individuals receiving definitive radiotherapy for NSCLC.

## Materials and Methods

### Study population

This retrospective analysis was approved by the institutional review board The University of Texas MD Anderson Cancer Center, and complied with all applicable Health Insurance Portability and Accountability Act regulations. Informed consents were wavered. Eligibility criteria were (1) receipt of definitive radiotherapy to a total radiation dose of ≥60 Gy [or ≥60 Gy (RBE) for proton therapy between 2006 and 2014 with chemotherapy for NSCLC; (2) histologically confirmed NSCLC from 1999 through 2014; and (3) available archived blood samples for genotyping. Patients who had received stereotactic ablative radiotherapy were excluded. A total of 664 patients met these criteria and were the subjects of this analysis.

### SNP selection and genotyping methods

Potentially functional and tagging SNPs were selected using https://snpinfo.niehs.nih.gov/snpinfo/snpfunc.html. The inclusion criteria were (1) having a minor allele frequency of >5% among whites and (2) being located in a transcription factor binding site, a microRNA‐binding site, or a nonsynonymous mutation in the coding area.

Genomic DNA's extract, evaluation, and storage have been described in our previous studies 14, 15. The primary extracted genomic DNA from blood samples was diluted into 5 ng/*μ*L aliquots for genotyping with TaqMan real‐time polymerase chain reaction. Primers and probes were from Applied Biosystems. For all genotypes, the assay success rate was >95%, and concordance of repeated sample testing was 100%.

### Statistical analyses

Potential associations between patient‐ and treatment‐related factors and outcomes, and potential associations between genotypes (genotype distribution was shown in Table S1) and outcomes, were assessed with a Cox proportional hazards model, with consideration of time to the event. Characteristics with *P*‐values<0.05 in the univariate Cox analysis were entered into the multivariate analysis; characteristics with *P‐*values of >0.20 in the multivariate analysis were removed. Only significant factors identified in the multivariate analyses were entered into the nomograms. Statistical significance levels were all two‐sided, with statistical significance set at 0.05.

## Results

### Patient characteristics

Characteristics of the study population are shown in Table 1. This study used the same database with our previous study 14, 16. The median age of the patients was 66 years (range 35–88 years), and most (488 [73.2%]) had stage III NSCLC. Radiation was delivered as proton beam therapy to 139 patients (20.8%), as intensity‐modulated (photon) radiotherapy to 331 (49.6%), and as three‐dimensional conformal radiotherapy to 174 (26.1%).

**Table 1:** Patient characteristics

Characteristics	No. patients (%)
Age, years	
<66 (median)	332 (50)
≥66	332 (50)
Sex	
Male	363 (55)
Female	301 (45)
Race	
White	569 (86)
Other	95 (14)
Disease stage	
I‐IIIA	296 (45)
IIIB, IV, recurrence	333 (50)
Unknown	35 (5)
Tumor histology	
Adenocarcinoma	293 (44)
SCC and Other	371 (56)
Karnofsky performance status score	
<80	101 (15)
≥80	563 (85)
Induction chemotherapy	
No	417 (63)
Yes	247 (37)
Smoking status	
Never	55 (8)
Former or current	597 (90)
Unknown	12 (2)
Total radiation dose, Gy	
<69.03 (median)	328 (49)
≥69.03	330 (50)
Unknown	6 (1)
Gross tumor volume, cm^3^	
<95.2 (median)	306 (46)
≥95.2	305 (46)
Unknown	53 (8)
Mean lung dose, Gy	
<17.9 (median)	320 (48)
≥17.9	319 (48)
Unknown	25 (4)
Radiation modality	
Photon (X‐ray)	511 (77)
Proton	139 (21)
Unknown	14 (2)

SCC, squamous cell carcinoma.

### Clinical factors, SNPs, and overall survival

Univariate analyses of patient‐ and treatment‐related factors for potential associations with OS were shown in Table S2. Factors with *P *<* *0.05 were entered into the multivariate analyses. Multivariate analyses revealed two factors associated with worse OS and two with better OS: Age ≥66 years (hazard ratio [HR]=1.247, 95% confidence interval [CI] 1.016–1.530, *P *=* *0.034) and GTV ≥95.2 cm^3^ (HR=1.890, 95% CI 1.539–2.321, *P *<* *0.001) were associated with poor OS. Conversely, receipt of concurrent chemotherapy (HR=0.706, 95% CI 0.515–0.968, *P *=* *0.031) and Karnofsky performance status score (KPS) ≥80 (HR=0.753, 95% CI 0.581–0.977, *P *=* *0.031) were associated with improved OS (Table 2).

**Table 2:** Multivariate Cox regression analyses for association between characteristics and disease outcome in patients with NSCLC receiving definitive radiotherapy

Characteristics	OS		PFS		LRRFS		DMFS	
	HR (95% CI)	*P*	HR (95% CI)	*P*	HR (95% CI)	*P*	HR (95% CI)	*P*
Age (≥66 vs. < 66)	1.247 (1.016–1.530)	0.034	NI		NI		0.853 (0.665–1.095)	0.213
Sex (male vs. female)	1.134 (0.922–1.396)	0.233	NI		NI		NI	
Race (black and other vs. white)	1.239 (0.952–1.612)	0.11	1.262 (0.945–1.685)	0.116	NI		NI	
Stage (IIIB, IV, recurrence vs. I‐IIIA)	NI		1.315 (1.432–2.215)	<0.001	NI		1.335 (1.041–1.713)	0.023
Histology (SCC and other vs. adeno)	1.160 (0.941–1.431)	0.164	NI		1.402 (1.040–1.889)	0.027	0.722 (0.561–0.928)	0.011
KPS (≥80 vs. < 80)	0.753 (0.581–0.977)	0.032	NI		NI		NI	
Concurrent chemotherapy (yes vs.no)	0.706 (0.515–0.968)	0.031	NI		NI		NI	
Smoking status (current/former vs. never)	NI		NI		NI		NI	
Total radiation dose (≥69.03 vs. <69.03 Gy)	NI		NI		NI		NI	
GTV (≥95.2 vs. <95.2 cm^3^)	1.890 (1.539–2.321)	<0.001	1.781 (1.432–2.215)	<0.001	1.317 (0.985–1.761)	0.063	1.951 (1.514–2.514)	<0.001
MLD (≥17.9 vs. <17.9 Gy)	NI		NI		NI		NI	
Technique (Proton vs. 3D‐CRT+IMRT)	NI		NI		NI		0.887 (0.647–1.214)	0.453

OS, overall survival; PFS, progression‐free survival; LRRFS, local‐regional recurrence‐free survival; DM, distant metastasis‐free survival; HR, hazard ratio; CI, confidence interval; NI, not included due to the *P‐*value >0.2; SCC, squamous cell carcinoma; adeno, adenocarcinoma; KPS, Karnofsky performance status score; GTV, gross tumor volume; MLD, mean lung dose; 3D‐CRT, three‐dimensional conformal (photon) radiation therapy; IMRT, intensity‐modulated (photon) radiation therapy.

Characteristics with a *P*‐value of <0.05 in the univariate analysis were entered into the multivariate model in a stepwise fashion and were removed if at any point the *P*‐value was >0.20.

Distributions of alleles for each SNP were shown in Table S1. Univariate analysis identified two significant SNPs associated with OS, as shown in Table S3. Analysis of potential associations of OS with the 13 analyzed SNPs revealed two SNPs were significant in multivariate analysis (Table 3): *Hamp* rs1882694 (poor OS for AC/CC vs. AA: HR = 1.301, 95% CI 1.040–1.627, *P *=* *0.021) and *Hamp* rs10421768 (poor OS for AG/GG vs. AA: HR = 1.253, 95% CI 1.015–1.546, *P *=* *0.035).

**Table 3:** Multivariate analysis of associations between single nucleotide polymorphisms and disease outcome in patients with NSCLC receiving definitive radiotherapy

SNPs	OS		PFS		LRRFS		DMFS	
	HR (95% CI)	*P*	HR (95% CI)	*P*	HR (95% CI)	*P*	HR (95% CI)	*P*
Hamp								
rs1882694 (AC/CC vs. AA)	1.301 (1.040–1.627)	0.021	1.435 (1.127–1.827)	0.003	1.648 (1.188–2.286)	0.003	1.348 (1.025–1.773)	0.033
rs10421768 (AG/GG vs. AA)	1.253 (1.015–1.546)	0.035	1.337 (1.066–1.677)	0.012	1.423 (1.054–1.922)	0.021	1.282 (0.993–1.655)	0.057
rs10402233 (AG/AA vs. GG)	1.040 (0.765–1.414)	0.801	0.966 (0.683–1.366)	0.844	0.654 (0.434–0.986)	0.042	1.005 (0.679–1.486)	0.981
rs12971321 (GC/GG vs. CC)	1.032 (0.771–1.380)	0.843	1.105 (0.808–1.511)	0.533	1.053 (0.695–1.596)	0.807	1.221 (0.844–1.765)	0.289
BMP2								
rs170986 (AC/CC vs. AA)	0.951 (0.576–1.570)	0.843	1.203 (0.666–2.171)	0.54	1.088 (0.510–2.322)	0.826	1.092 (0.558–2.137)	0.798
rs1979855 (AG/GG vs. AA)	1.189 (0.950–1.489)	0.13	1.433 (1.128–1.820)	0.003	1.433 (1.042–1.970)	0.027	1.259 (0.958–1.654)	0.098
rs3178250 (CT/TT vs. CC)	0.671 (0.391–1.153)	0.149	0.370 (0.207–0.663)	0.001	1.035 (0.383–2.800)	0.946	0.356 (0.187–0.678)	0.002
rs1980499 (CT/TT vs. CC)	1.116 (0.869–1.434)	0.389	1.319 (1.009–1.725)	0.043	1.318 (0.927–1.873)	0.125	1.260 (0.934–1.700)	0.13
rs235768 (AT/TT vs. AA)	1.195 (0.882–1.618)	0.25	1.226 (0.898–1.674)	0.2	1.101 (0.741–1.638)	0.633	1.285 (0.896–1.843)	0.173
BMP4								
rs4898820 (GT/TT vs. GG)	1.165 (0.895–1.516)	0.257	1.130 (0.855–1.494)	0.391	0.964 (0.676–1.376)	0.84	1.225 (0.886–1.695)	0.219
rs762642 (AC/AA vs. CC)	1.044 (0.784–1.391)	0.766	1.107 (0.813–1.506)	0.518	1.123 (0.745–1.693)	0.58	1.223 (0.856–1.746)	0.268
rs17563 (AG/GG vs. AA)	1.022 (0.797–1.312)	0.862	0.956 (0.736–1.241)	0.734	0.954 (0.684–1.331)	0.782	0.945 (0.714–1.253)	0.695
Smad4								
rs12456284 (AG/AA vs. GG)	0.829 (0.557–1.232)	0.353	0.739 (0.491–1.114)	0.149	0.882 (0.491–1.585)	0.674	0.745 (0.476–1.166)	0.198

OS, overall survival; PFS, progression‐free survival; LRRFS, local‐regional recurrence‐free survival; DMFS, distant metastasis‐free survival; HR, hazard ratio; CI, confidence interval.

**Figure 1:**
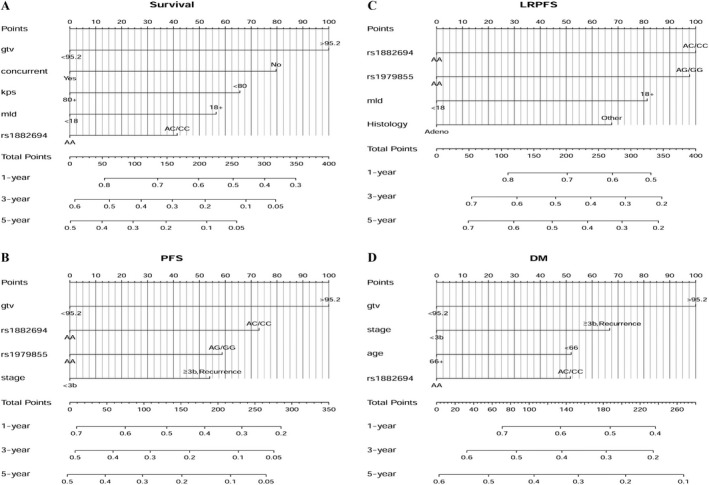
(A) Nomogram for overall survival. Abbreviations: KPS, Karnofsky performance status score; GTV, gross tumor volume; MLD, mean lung dose. (B) Nomogram for progression‐free survival. Abbreviations: GTV, gross tumor volume. (C) Nomogram for local‐regional progression‐free survival. Abbreviations: MLD, mean lung dose. (D) Nomogram for distant metastasis‐free survival. Abbreviations: GTV, gross tumor volume.

The model that incorporated the independent predictors identified in the multivariate analyses (Tables 2 and 3) was developed and presented as the nomogram (Fig. 1A). For example, a patient was with GTV = 100 cm^3^, KPS = 90, mld = 19 Gy, *Hamp* rs1882694 CC genotypes and received concurrent chemoradiation, the total points would be 197 (points for each factor: GTV = 100, concurrent chemoradiation = 0, KPS = 0, MLD = 56, CC genotypes = 41, total points = 100 + 0 + 0 + 56 + 41 = 197). The one‐year survival probability would be 60%; the three‐year survival probability would be 22%, and the five‐year survival probability would be 11%. Likelihood ratio (LR) was used to compare the predictive capacities between different models. The prediction model yielded an LR of 54.22 with SNP and 50.55 without SNP. Adding the SNP into the nomogram improved the prediction of OS marginally (*P *=* *0.056, Table 4)

**Table 4:** Likelihood ratio for each endpoint with or without SNPs

	With SNP		Without SNP		Increase with SNP		
	LR	df	LR	df	Chi‐square	df	*P*‐value
OS	54.22	5	50.55	4	3.66	1	0.056
PFS	38.44	4	24.37	2	14.07	2	0.001
LRPFS	25.01	4	10.54	2	14.46	2	0.001
DMFS	31.63	4	27.99	3	3.64	1	0.057

OS, overall survival; PFS, progression‐free survival; LRPFS, local‐regional progression‐free survival; DMFS, distant metastasis‐free survival; LR, likelihood ratio; df, degree of freedom.

### Clinical factors, SNPs, and progression‐free survival

Not surprisingly, higher disease stage and larger tumor volume were strongly associated with poor PFS, in both univariate analysis (stage: HR = 1.400, 95% CI 1.141–1.718, *P *=* *0.001; GTV: HR = 1.911, 95% CI 1.563–2.336, *P *<* *0.001, Table S2) and multivariate analysis (stage: HR = 1.315, 95% CI 1.432–2.215, *P *<* *0.001; GTV: HR = 1.890, 95% CI 1.539–2.321, *P *<* *0.001, Table 2).

In univariate analysis, seven SNPs associated with PFS (Table S3); however, five SNPs held their significance in multivariate analyses: two SNPs in *Hamp* (rs1882694, AC/CC vs. AA: HR = 1.435, 95% CI 1.127–1.827, *P *=* *0.003; and rs10421768, AG/GG vs. AA: HR = 1.337, 95% CI 1.066–1.677, *P *=* *0.012) and three SNPs in *BMP2* (rs1979855, AG/GG vs. AA: HR = 1.433, 95% CI 1.128–1.820, *P *=* *0.003; rs3178250, CT/TT vs. CC: HR = 0.370, 95% CI 0.207–0.663, *P *=* *0.001; and rs1980499, CT/TT vs. CC: HR = 1.319, 95% CI 1.009–1.725, *P *=* *0.043) (Table 3).

Significant factors of the multivariate analyses were included to establish the predictive nomogram (Fig. 1B). As shown in Table 4, the LR of the model with SNPs (LR = 38.44) was increased significantly (*P *=* *0.001), compared to the model without SNPs (LR = 24.37). The significant SNPs could obviously improve the capacity of the nomogram to predict the PFS.

### Clinical factors, SNPs, and local‐regional progression‐free survival

In the analysis of clinical factors and LRPFS, three factors (histology, GTV, and MLD) were linked with LRPFS in univariate analyses (Table S2), but only histology remained significant in multivariate analysis (squamous cell carcinoma and other vs. adenocarcinoma: HR = 1.403, 95% CI 1.056–1.864, *P *=* *0.020) (Table 2).

A total of four SNPs were associated with LRFFS in univariate analyses (Table S3). These four SNPs were also statistically significant in multivariate analyses: three in *Hamp* (rs1882694, AC/CC vs. AA: HR = 1.648, 95% CI 1.188–2.286, *P *=* *0.003; rs10421768, AG/GG vs. AA: HR = 1.423, 95% CI 1.054–1.922, *P *=* *0.021; and rs10402233, AG/AA vs. GG: HR = 0.654, 95% CI 0.434–0.986, *P *=* *0.042) and one in *BMP2* (rs1979855, AG/GG vs. AA: HR = 1.433, 95% CI 1.042–1.970, *P *=* *0.027) (Table 3).

Histology, MLD, rs1882694, and rs1979855 were used to develop nomogram (Fig. 1C). One‐year LRPFS, 3‐year LRPFS, and 5‐year LRPFS could be estimated through our established nomograms. Patients with risk genotypes (*Hamp* rs1882694 AC/CC and *BMP2* rs1979855 AG/GG), higher MLD, and nonadenoma histology had poor LRPFS. The LR was increased significantly after adding the SNPs into the nomogram (LR = 25.01, *P *=* *0.001), compared to without SNPs (LR = 10.54). (Table 4).

### Clinical factors, SNPs, and distant metastasis

Three clinical factors were associated with DMFS on multivariate analysis: disease stage (IIIB, IV, recurrence vs. I–IIIA: HR = 1.335, 95% CI 1.041–1.713, *P *=* *0.023); histology (squamous and other vs. adenocarcinoma: HR = 0.722, 95% CI 0.561–0.928, *P *=* *0.011); and GTV (≥95.2 vs. <95.2 cm^3^: HR = 1.951, 95% CI 1.514–2.514, *P *<* *0.001) (Table 2).

Five SNPs were associated significantly with DMFS in univariate analyses (Table S3). Finally, two SNPs were found to be associated with DMFS in multivariate analyses (Table 3), one in *Hamp* (rs1882694, AC/CC vs. AA: HR = 1.348, 95% CI 1.025–1.773, *P *=* *0.033) and one in *BMP2* (rs3178250, CT/TT vs. CC: HR = 0.356, 95% CI 0.187–0.678, *P *=* *0.002).

Based on the data above, disease stage, histology, GTV, and rs1882694 were considered as independent factors for DMFS to develop the nomogram (Fig. 1D). Without SNP, the LR of the model was 27.99, and the LR (31.63) was increased marginally after adding the SNP (*P *=* *0.057) (Table 4). The high‐risk factors of DM included greater GTV, advanced disease stage, younger age, and *Hamp* rs1882694 AC/CC genotypes.

## Discussion

In the current study, we found that SNPs in hepcidin regulatory BMP/Smad4/Hamp pathway were significantly associated with disease outcomes after definitive radiotherapy in NSCLC; *Hamp* rs1882694 in particular was associated with all of the studied endpoints in both univariate and multivariate analyses. As far as we know, this is the first study to investigate associations between genetic variants in hepcidin regulatory pathway and prognosis for patients with NSCLC after definitive radiotherapy. Nomograms established in our study will be helpful to predict individual outcome in such patients.

*Hamp* is the gene that codes for hepcidin. Increased expression of hepcidin has been thought to indicate unfavorable outcomes for patients with various malignancies including breast cancer, prostate cancer, renal cell carcinoma, and lymphoma 6, 9, 10, 17, 18. Similar results were found in our preliminary data (in review). Previous studies of genetic variants in hepcidin or hepcidin regulation pathways focused on iron burden; for example, one group found common variants in hepcidin regulation pathways to be associated with penetrance of HFE (hereditary) hemochromatosis penetrance 19, and another group implicated variants in *Hamp* regulators as well 20.

Our results can be explained from a biological standpoint. Hepcidin, secreted by hepatocytes, has a crucial role in iron homeostasis. The major role of hepcidin is to regulate the surface expression of ferroportin 1 (FPN1), the only known iron‐exporting protein; after hepcidin binds with FPN1 and thereby induces the degradation FPN1 21, enterocytes, macrophages, and hepatocytes can no longer export iron. Similar phenomenon can also be observed. The expression of hepcidin could promote the cancer cell survival by inducing the iron retentions in tumor cells 8. High expression of hepcidin decreases plasma iron concentrations; low expression increases iron concentrations 22. Hepcidin expression is upregulated by high concentrations of iron in the liver and plasma, inflammation, and physical activity 23. Radiation‐induced expressions of IL‐1 24 and IL‐6 25 could promote synthesis of hepcidin, which would be accompanied by a reduced availability of iron. Deficiency in iron in turn leads to anemia, which is thought to result in hypoxia‐induced resistance to radiation in tumor cells 26. Genetic variants in Hamp or Hamp regulation pathways thus might be expected to induce aberrant expression of hepcidin and therefore promote the proliferation and treatment resistance of tumor cells.

Our findings that SNPs in Hamp or Hamp regulation pathways could predict outcomes among patients with NSCLC after definitive radiotherapy have several implications for tailoring treatment, moving from “one size fits all” to individualized therapy. Our nomograms could help better identify patients with poor outcomes. For instance, if patients with high‐risk alleles were more likely to develop DM, closer follow‐up would be recommended. Both previous findings in breast cancer and our results shed light on the roles of iron metabolism in tumor progression and implicate potential targets for therapy. Examples of such strategies may include depleting hepcidin using neutralizing antibodies, hepcidin small‐interfering RNAs, or microRNAs, or by inhibiting pathways that stimulate hepcidin expression, such as CEBPa or CEBPb 27, 28, 29, 30.

Strengths of our study were the relatively large number of patients analyzed (*n *=* *664) and its rigorous design: Follow‐up was comprehensive and included information on OS, DMFS, LRPFS, and PFS. Further, clinical characteristics were evaluated carefully with regard to each observed endpoint, and so that factors that met the statistical requirements could be included in the subsequent multivariate model. This is also the first study to develop nomograms incorporating clinical characteristics and genetic background to predict OS, PFS, LRPFS, and DMFS.

Our study is limited by its retrospective and single‐institution nature. And we analyzed patients with a wide latency (1999–2014), and other kinds of treatment options have been rapidly developed in recent years. Thus, these findings require validation in studies of other patient populations at other institutions. We also did not investigate potential mechanistic explanations for our positive results. Finally, we studied only potentially functional and tagging SNPs, rather than all of the SNPs in the entire gene.

In conclusion, genetic variants in hepcidin regulation pathways help improve the prediction of outcomes among patients receiving definitive radiotherapy for NSCLC, especially rs1882694, which was predictive of all studied endpoints. It is necessary to validate our findings in patients receiving other types of treatments, for example, targeted therapies and immunotherapies.

## Conflict of Interest

None declared.

## Supporting information


**Table S1.** Genotype distribution of our studied SNPs.
**Table S2.** Univariate Cox regression analyses for association between characteristics and disease outcome in patients with NSCLC receiving definitive radiotherapy.
**Table S3.** Univariate analysis of associations between single‐nucleotide polymorphisms and disease outcome in patients with NSCLC receiving definitive radiotherapy.
