# Supplementary material for: Nomograms incorporating genetic variants in BMP/Smad4/Hamp pathway to predict disease outcomes after definitive radiotherapy for non‐small cell lung cancer
Source: Cancer Med. 2018 May 9;7(6):2247–55. doi: 10.1002/cam4.1349 (PMC6010922; doi:10.1002/cam4.1349)
Supplement: Supplementary file 1 — Table S1. Genotype distribution of our studied SNPs. Table S2. Univariate Cox regression analyses for association between characteristics and disease outcome in patients with NSCLC receiving definitive radiotherapy. Table S3. Univariate analysis of associations between single‐nucleotide polymorphisms and disease outcome in patients with NSCLC receiving definitive radiotherapy. [file CAM4-7-2247-s001.docx]

**Table S1** Genotype distribution of our studied SNPs

| Genes | SNPs |  |  |  |  |
| --- | --- | --- | --- | --- | --- |
| Hamp | Rs1882694 | AA | 221 | AC/CC | 378 |
|  | Rs10421768 | AA | 412 | AG/GG | 229 |
|  | Rs10402233 | GG | 74 | AG/AA | 563 |
|  | Rs12971321 | CC | 98 | GC/GG | 540 |
| BMP2 | Rs170986 | AA | 25 | AC/CC | 615 |
|  | Rs1979855 | AA | 468 | AG/GG | 171 |
|  | Rs3178250 | CC | 20 | CT/TT | 625 |
|  | Rs1980499 | CC | 162 | CT/TT | 477 |
|  | Rs235768 | AA | 106 | AT/TT | 534 |
| BMP4 | Rs4898820 | GG | 128 | GT/TT | 505 |
|  | Rs762642 | CC | 97 | AC/AA | 528 |
|  | Rs17563 | AA | 167 | AG/GG | 475 |
| Smad4 | Rs12456284 | GG | 46 | AG/AA | 596 |

**Table S2:** Univariate Cox regression analyses for association between characteristics and disease outcome in patients with NSCLC receiving definitive radiotherapy

| **Characteristics** | **OS** |  |  | **PFS** |  |  | **LRRFS** |  |  | **DMFS** |  |
| --- | --- | --- | --- | --- | --- | --- | --- | --- | --- | --- | --- |
|  | **HR (95% CI)** | ***P*** |  | **HR (95% CI)** | ***P*** |  | **HR (95% CI)** | ***P*** |  | **HR (95% CI)** | ***P*** |
| Age (≥66 vs.<66) | 1.334 (1.105–1.612) | 0.003 |  | 0.876 (0.718-1.069) | 0.194 |  | 1.086 (0.824–1.431) | 0.558 |  | 0.793 (0.633–0.994) | 0.044 |
| Sex (male vs. female) | 1.279 (1.058–1.545) | 0.011 |  | 1.151 (0.943-1.405) | 0.167 |  | 1.196 (0.907–1.578) | 0.205 |  | 1.049 (0.838–1.313) | 0.677 |
| Race (black and other vs. white) | 1.284 (0.999–1.651) | 0.051 |  | 1.262 (0.958-1.662) | 0.099 |  | 1.226 (0.840–1.788) | 0.291 |  | 1.260 (0.928–1.712) | 0.139 |
| Stage (IIIB, IV, recurrence vs. I-IIIA) | 1.056 (0.873–1.278) | 0.575 |  | 1.400 (1.141-1.718) | 0.001 |  | 1.130 (0.821–1.499) | 0.398 |  | 1.449 (1.151–1.826) | 0.002 |
| Histology (SCC and other vs. adeno) | 1.310 (1.081–1.588) | 0.006 |  | 0.991 (0.811-1.210) | 0.926 |  | 1.403 (1.056–1.864) | 0.02 |  | 0.829 (0.662–1.038) | 0.102 |
| KPS (≥80 vs.<80) | 0.666 (0.508–0.861) | 0.002 |  | 1.084 (0.801-1.466) | 0.603 |  | 0.894 (0.600–1.333) | 0.584 |  | 1.051 (0.748–1.477) | 0.774 |
| Concurrent chemotherapy (yes vs.no) | 1.153 (0.808–1.65) | 0.432 |  | 1.030 (0.745-1.421) | 0.856 |  | 1.053 (0.664–1.671) | 0.826 |  | 0.950 (0.769–1.654) | 0.538 |
| Smoking status (current/former vs. never) | 1.153 (0.808–1.645) | 0.432 |  | 1.056 (0.731-1.525) | 0.771 |  | 1.250 (0.726–2.153) | 0.421 |  | 0.950 (0.635–1.421) | 0.803 |
| Total radiation dose (≥69.03 vs. <69.03 Gy) | 0.871 (0.721–1.052) | 0.153 |  | 0.878 (0.719-1.027) | 0.201 |  | 0.958 (0.725–1.265) | 0.762 |  | 0.847 (0.677–1.060) | 0.147 |
| GTV (≥95.2 vs. <95.2 cm^3^) | 1.911 (1.563–2.336) | <0.001 |  | 1.758 (1.424-2.169) | <0.001 |  | 1.383 (1.037–1.843) | 0.027 |  | 1.897 (1.496–2.406) | <0.001 |
| MLD (≥17.9 vs. <17.9 Gy) | 1.532 (1.262–1.860) | <0.001 |  | 1.477 (1.203-1.813) | <0.001 |  | 1.422 (1.072–1.886) | 0.014 |  | 1.479 (1.173–1.865) | 0.001 |
| Technique (Proton vs. 3D-CRT+IMRT) | 0.825 (0.646–1.052) | 0.121 |  | 0.930 (0.728-1.190) | 0.566 |  | 1.080 (0.778–1.501) | 0.644 |  | 0.766 (0.573–1.025) | 0.073 |

Note: Characteristics with a *P* value of <0.05 in the univariate analysis were entered into the multivariate model in a stepwise fashion and were removed if at any point the *P* value was >0.20.

Abbreviations: OS, overall survival; PFS, progression free survival; LRRFS, local regional recurrence free survival; DM, distant metastasis free survival; HR, hazard ratio; CI, confidence interval; NI, not included; SCC, squamous cell carcinoma; adeno, adenocarcinoma; KPS, Karnofsky Performance Status score; GTV, gross tumor volume; MLD, mean lung dose; 3D-CRT, 3-dimensional conformal (photon) radiation therapy; IMRT, intensity-modulated (photon) radiation therapy.

**Table S3**. Univariate analysis of associations between single-nucleotide polymorphisms and disease outcome in patients with NSCLC receiving definitive radiotherapy

| **SNPs** | **OS** | |  | **PFS** | |  | **LRRFS** | |  | **DMFS** | |
| --- | --- | --- | --- | --- | --- | --- | --- | --- | --- | --- | --- |
|  | **HR (95% CI)** | ***P*** |  | **HR (95% CI)** | ***P*** |  | **HR (95% CI)** | ***P*** |  | **HR (95% CI)** | ***P*** |
| **Hamp** |  |  |  |  |  |  |  |  |  |  |  |
| rs1882694 (AC/CC vs. AA) | 1.251 (1.017–1.539) | 0.034 |  | 1.345 (1.079–1.676) | 0.008 |  | 1.609 (1.176–2.201) | 0.003 |  | 0.733 (0.542–0.992) | 0.044 |
| rs10421768 (AG/GG vs. AA) | 1.181 (0.968–1.441) | 0.101 |  | 1.238 (1.002–1.529) | 0.048 |  | 1.378 (1.032–1.840) | 0.030 |  | 1.222 (0.964–1.548) | 0.098 |
| rs10402233 (AG/AA vs. GG) | 0.889 (0.668–1.183) | 0.418 |  | 0.899 (0.656–1.232) | 0.509 |  | 0.666 (0.445–0.995) | 0.047 |  | 0.921 (0.647–1.312) | 0.649 |
| rs12971321 (GC/GG vs. CC) | 1.050 (0.801–1.375) | 0.725 |  | 1.097 (0.824–1.461) | 0.525 |  | 0.771 (0.462–1.287) | 0.32 |  | 1.171 (0.842–1.629) | 0.349 |
| **BMP2** |  |  |  |  |  |  |  |  |  |  |  |
| rs170986 (AC/CC vs. AA) | 0.868 (0.541–1.392) | 0.557 |  | 1.095 (0.629–1.904) | 0.749 |  | 0.948 (0.703–1.279) | 0.727 |  | 1.037 (0.552–1.950) | 0.909 |
| rs1979855 (AG/GG vs. AA) | 1.276 (1.035–1.574) | 0.023 |  | 1.492 (1.196–1.860) | <0.001 |  | 1.397 (1.026–1.901) | 0.034 |  | 1.346 (1.047–1.729) | 0.020 |
| rs3178250 (CT/TT vs. CC) | 1.312 (0.997–1.725) | 0.052 |  | 0.465 (0.267–0.810) | 0.007 |  | 0.963 (0.358–2.594) | 0.941 |  | 0.453 (0.247–0.828) | 0.010 |
| rs1980499 (CT/TT vs. CC) | 1.200 (0.985–1.505) | 0.113 |  | 1.422 (1.117–1.810) | 0.004 |  | 1.337 (0.957–1.867) | 0.089 |  | 1.350 (1.028–1.773) | 0.031 |
| rs235768 (AT/TT vs. AA) | 1.312 (0.997–1.725) | 0.052 |  | 1.352 (1.021–1.790) | 0.035 |  | 1.074 (0.741–1.557) | 0.706 |  | 1.401 (1.014–1.937) | 0.041 |
| **BMP4** |  |  |  |  |  |  |  |  |  |  |  |
| rs4898820 (GT/TT vs. GG) | 1.004 (0.787–1.280) | 0.974 |  | 1.000 (0.777–1.286) | 0.999 |  | 0.904 (0.642–1.273) | 0.564 |  | 1.051 (0.787–1.403) | 0.736 |
| rs762642 (AC/AA vs. CC) | 1.016 (0.777–1.329) | 0.909 |  | 0.783 (0.637–0.962) | 0.020 |  | 1.061 (0.714–1.575) | 0.771 |  | 1.041 (0.757–1.431) | 0.806 |
| rs17563 (AG/GG vs. AA) | 0.874 (0.705–1.082) | 0.216 |  | 0.859 (0.684–1.079) | 0.191 |  | 0.961 (0.697–1.326) | 0.809 |  | 0.892 (0.689–1.154) | 0.383 |
| **Smad4** |  |  |  |  |  |  |  |  |  |  |  |
| rs12456284 (AG/AA vs. GG) | 1.036 (0.845–1.269) | 0.736 |  | 0.756 0.523–1.092) | 0.136 |  | 1.158 (0.855–1.569) | 0.344 |  | 0.771(0.516–1.154) | 0.206 |

Abbreviations: OS, overall survival; PFS, progression free survival; LRRFS, local regional recurrence free survival; DM, distant metastasis free survival; HR, hazard ratio; CI, confidence interval.
